# Seasonal Variation in Leaf Mineral Nutrients and the Determination of the Nutritional Diagnostic Period of *Paeonia ostii*

**DOI:** 10.3390/plants15121884

**Published:** 2026-06-17

**Authors:** Yu Duan, Wei Zhao, Chen Zhang, Li Chen, Liyong Sun, Shuxian Li

**Affiliations:** 1 College of Forestry and Grass, Nanjing Forestry University, Nanjing 210037, China; duanyu@njfu.edu.cn (Y.D.);; 2 Southern Modern Forestry Collaborative Innovation Center, Nanjing Forestry University, Nanjing 210037, China; 3 State Key Laboratory of Tree Genetics and Breeding, Nanjing 210037, China; 4 Department of Architecture and Environmental Engineering, Wuxi City College of Vocational Technology, Wuxi 214153, China

**Keywords:** mineral nutrition, nutrient diagnosis, *Paeonia ostii*, plant growth, scientific fertilisation

## Abstract

*Paeonia ostii*, a significant perennial woody oil crop in China, is notable for its seeds’ high oil content and elevated levels of unsaturated fatty acids. However, there is currently a lack of scientific fertilisation protocols and targeted nutrient management for *P. ostii*. The concentrations of macronutrients (N, P, K, Ca, and Mg) and micronutrients (Fe, Mn, Zn, and Cu) were determined in the leaves at five distinct growth stages: flowering, initial fruit set, fruit expansion, late fruiting, and foliar senescence. The levels of N and P were found to be at their highest point during the flowering stage, after which they declined significantly. In contrast, the levels of K remained relatively stable throughout the growth phase, while Mg levels increased significantly to peak at fruit expansion. The level of Ca increased, reaching its peak at the late fruiting stage. The annual average content of micronutrients in *P. ostii* leaves was as follows: Fe > Mn > Zn > Cu. Furthermore, it was observed that the concentrations of Fe and Mn oscillated, while the concentration of Cu decreased significantly after flowering. Additionally, Zn concentrations remained stable throughout the various stages. Multivariate analyses, including PCA, nutrient ratio analysis, and an integrated nutrient stability index, further revealed coordinated shifts in leaf nutrient composition and indicated that May and June were relatively stable periods for nutrient assessment. Considering both the nutrient stability and the phenological relevance, June, corresponding to the fruit expansion stage, was considered a practical sampling window for foliar nutrient diagnosis. These findings contribute to the definition of an appropriate sampling window for foliar nutrient diagnosis, thereby providing a useful basis for nutrient monitoring and future fertilisation studies in *P. ostii*.

## 1. Introduction

*Paeonia ostii,* a perennial deciduous shrub in the genus *Paeonia*, is a renowned traditional woody flower unique to China for its excellent ornamental characteristics [1]. The plant has become a popular commercial crop worldwide, primarily due to its attractive appearance and the wide variety of available cultivars [2]. In the domain of traditional Chinese medicine (TCM), the root bark, known as mudanpi, has a long history of utilization in the treating of cardiovascular diseases, stagnant blood, stagnated blood, and female genital diseases [3]. Recently, *P. ostii* seeds have become a popular source of desirable woody oilseed plants thanks to their high levels of unsaturated fatty acids (UFA: >90%) and alpha-linolenic acid (ALA: >40%) [2,4,5,6].

Fertilisation exerts a profound influence on plant growth and development, serving as an important strategy for enhancing yield and improving product quality [7]. However, research on precision fertilisation regimes for *P. ostii* remains notably scarce. In actual production, the excessive application of fertiliser is a common occurrence, resulting in fertiliser wastage and an imbalanced nutrient uptake by the plant [8]. The phenomenon may also result in significant complications, including the processes of acidification and salinisation of the soil microenvironment [9]. Fertiliser misuse poses a considerable challenge to sustainable agriculture. Diagnosing plant nutrient deficiencies offers a targeted and effective means of addressing this issue. The method involves the evaluation of the nutritional status of plants through the analysis of the content and proportions of nutrients present in the leaf. In recent years, this analysis has been regarded as a valuable tool for determining fertiliser requirements [10]. As is evident in the extant literature, the diagnosis of leaf nutrient status enables the identification of nutrient deficiency, excess, or imbalance. This, in turn, facilitates the determination of precise fertiliser application [11]. The establishment of a stable and representative sampling period is imperative for the accurate diagnosis of nutritional status, as well as for the future recommendations regarding fertiliser use.

The nutrient concentration of foliage is subject to the influence of various factors, including the specific plant species, the nutrient availability within the soil, and the manner in which nutrients are distributed among the various plant organs. As is evidenced by the extant literature, different species differ in terms of their nutrient absorption, nutrient-use efficiency, and allocation strategies. This, in turn, results in variations in leaf nutrient composition [12]. As mineral nutrients are primarily absorbed from the soil by roots and subsequently transported within the plant, leaf nutrient status is closely related to soil nutrient supply [13]. Furthermore, the accumulation of nutrients may vary among different plant parts, including roots, stems, leaves, flowers, fruits, and seeds [14]. Despite the prevalence of indicators such as the bioconcentration factor (BCF) and translocation factor (TF) in the context of soil-to-plant accumulation and root-to-shoot transport in element accumulation studies [15], the present study concentrated on seasonal changes in leaf nutrient concentrations as a basis for foliar nutrient diagnosis.

It has been previously established that the most opportune moment for the purpose of leaf nutrient diagnosis is to be scheduled at a time when there is minimal variation in nutrient content and minor deviation from the annual average, alongside vigorous plant growth [16]. For example, Tian et al. suggested that the timing of nutrient diagnosis of *Boehmeria nivea* leaves should be conducted during the stage of rapid vegetative growth [17]. In their seminal study, Mo et al. proposed the hypothesis that the optimal period for diagnosing mineral nutrients in *Camellia oleifera* leaves is from June to August, when the morphological characteristics of the leaves are stable and the nutrient content is also relatively stable [18]. Zhuang’s report indicated that the optimal time for diagnosing nutrients in *Syzygium samarangense* is during the flowering stage [19]. In a similar vein, Li et al. discovered that the optimal time for diagnosing nutrients in *Oryza sativa* is during the booting stage [20]. The above research findings indicate that the optimal timing for diagnosing the nutritional status of various plant species varies significantly.

The annual growth and development process of *P. ostii* can be divided into the following key stages: budding in mid-March, flowering in early to mid-April, fruiting stage from May to early August, and the beginning of defoliation in mid-August. In this study, *P. ostii* leaves were collected in the middle of each month from April to August to determine the concentrations of nine mineral nutrients. The objective of this study was to characterize the seasonal variation patterns of leaf mineral nutrients and to evaluate the suitability of different phenological stages for foliar nutrient diagnosis. In addition to the coefficient of variation (CV) and annual mean deviation based scoring approach, nutrient ratios, principal component analysis, hierarchical clustering heatmaps, and an integrated nutrient stability index were used to assess nutrient balance, multivariate nutrient patterns, and sampling-period stability. This study aimed to ascertain a suitable sampling window for foliar nutrient diagnosis in *P. ostii*, with a view to provide a preliminary basis for nutrient monitoring and future fertilisation studies.

## 2. Materials and Methods

### 2.1. Experimental Site and Soil

The experiment is situated at the Baima Base of Nanjing Forestry University, which is located between 118°51′ and 119°14′ E and 31°23′ and 31°48′ N. This region has a subtropical monsoon climate in the north, featuring four distinct seasons: hot, and humid summers with frequent rainfall, and cold and dry winters. In 2023, the mean annual maximum and minimum annual temperatures were recorded at 22 °C and 12 °C, respectively, with a mean relative humidity of 76%.

The total annual precipitation was approximately 1160 mm, distributed across 100 rainy days. The fundamental physicochemical characteristics of the soil at the experimental site are outlined below: porosity, 37.83%; bulk density, 1.43 g/cm^3^; pH, 5.85; total N, 0.64 g/kg; total K, 17.93 g/kg; total P, 0.24 g/kg; available P, 7.91 mg/kg; and available K, 120.00 mg/kg.

The fertilisation process was conducted in accordance with the recommended application rate by Wei et al. [21]. The planting spacing was 70.00 cm × 100.00 cm, corresponding to a planting density of approximately 14,286 plants/ha. A total of 45.00 g of compound fertiliser (Stanley^TM^ Compound Fertiliser with an N-P-K content of 17-17-17, Stanley Agriculture Group Co., Ltd., Linyi, Shandong, China) was applied per plant, equivalent to approximately 642.86 kg/ha. The fertiliser was applied in three separate applications: in early October 2022 (root growth stage), in early March 2023 (before leaf expansion), and in early May 2023 (initial fruit stage). The respective proportions of these splits were 40%, 30%, and 30% of the total fertiliser amount. The study aimed to characterise seasonal variation in leaf nutrient concentrations under uniform field management conditions, consequently, no additional fertilisation treatments or separate control plots were established.

### 2.2. Plant Materials

**Figure 1:**
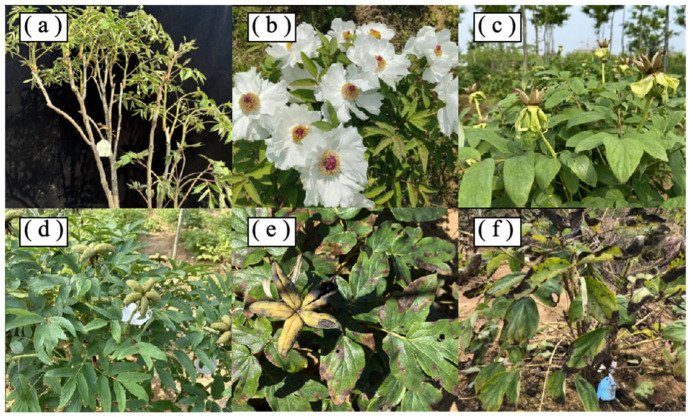
Photos of *P. ostii* during different development stages: (**a**) the budding stage; (**b**) the full flowering stage; (**c**) the early fruiting stage; (**d**) the fruit expansion stage; (**e**) the late fruiting stage; (**f**) the foliar senescence stage.

Eight-year-old *P. ostii* plants that were at full fruiting capacity and possessed a uniform canopy structure (height: 90 cm; canopy diameter: 100 cm) were selected at Baima Base. As the young leaves sampled at bud break are incompletely expanded and exhibit significant variations in nutrient concentrations (Figure 1a), fully expanded foliage was sampled instead. Leaves of *P. ostii* were collected at mid-month intervals from April to August 2023, coinciding with five critical phenological phases: 12 April (flowering stage, Figure 1b), 19 May (initial fruit set, Figure 1c), 15 June (fruit expansion, Figure 1d), 12 July (late fruit development, Figure 1e), and 15 August (early foliar senescence stage, Figure 1f).

At each designated time point, three healthy, pest- and disease-free plants were randomly selected for sampling [22], with each selected plant bearing at least seven fruiting branches. From each plant, four branches from the east, west, south, and north sides of the canopy were collected, and the second pair of leaflets from the second compound leaf below the shoot apex was sampled. A composite sample was formed by combining leaves collected from the four directions of the same plant. Each composite sample was designated as one biological replicate, thus yielding three biological replicates for each phenological stage. Since leaves were sampled at five distinct phenological stages, a total of 15 composite biological samples were analysed.

### 2.3. Nutritional Elements Measurement

The sampled leaflets were oven-dried at 65 °C for 72 h in a forced air dryer and then ground until they passed through a stainless steel sieve. The total N content was determined by the Kjeldahl method following the digestion of 0.2 g of dry powder with concentrated H_2_SO_4_ [23]. For the elements P, K, Ca, Mg, Cu, Fe, Mn, and Zn, 1.0 g of dry material was digested in a mixture of nitric acid and perchloric acid [24]. P content was measured in accordance with the vanadomolybdate method [25]. The quantification of K, Ca, Mg, Fe, Cu, Zn, and Mn was conducted using flame atomic absorption spectroscopy (PerkinElmer AA900T, Shelton, CT, USA) [26].

### 2.4. Diagnostic Period Scoring and Integrated Nutrient Stability Assessment

Following Zhang’s methodology [27], the coefficients of variation (CV) and absolute deviations from the annual mean for nutrients at different growth stages were systematically ranked and scored to ascertain diagnostic periods. The CV was used to evaluate within-period variability among biological replicates, whereas the deviation from the annual mean was used to assess the representativeness of each sampling period.

For each nutrient element, the CV and the absolute deviation from the annual mean were ranked among the five sampling months. The sampling month with the lowest CV or the smallest deviation from the annual mean received the highest score, whereas the month with the highest CV or the largest deviation received the lowest score. The two scores were then summed to obtain a comprehensive score for each nutrient element and sampling month. Higher comprehensive scores indicate greater temporal stability and stronger representativeness of the corresponding sampling period.

To further integrate nutrient stability and representativeness, a nutrient stability index (NSI) was calculated as an exploratory metric. For nutrient i in sampling month j, the coefficient of variation was calculated as:

(1) $${CV}_{ij}=\frac{{SD}_{ij}}{{Mean}_{ij}}$$

The relative absolute deviation from the annual mean was calculated as:

(2) $${RD}_{ij}=\frac{{|Mean}_{ij}-{AnnualMean}_{i}|}{{AnnualMean}_{i}}$$

where RD_ij_ represents the relative absolute deviation of nutrient i in month j, while Mean_ij_ and AnnualMean_i_ represent the monthly mean and annual mean concentrations of nutrient i, respectively.

Because lower CV and RD values indicate greater stability and representativeness, respectively, both indices were transformed into normalized scores ranging from 0 to 1 within each nutrient using min–max normalization:

(3) $${CVscore}_{ij}=1-\frac{{CV}_{ij}-{CV}_{i,min}}{{CV}_{i,max}-{CV}_{i,min}}$$

(4) $${RDscore}_{ij}=1-\frac{{RD}_{ij}-{RD}_{i,min}}{{RD}_{i,max}-{RD}_{i,min}}$$

where CV_i,min_ and CV_i,max_ represent the minimum and maximum CV values of nutrient i across the five sampling months, respectively, and RD_i,max_ and RD_i,min_ represent the maximum and minimum RD values of nutrient i across the five sampling months, respectively.

The NSI for each nutrient and sampling month was then calculated as:

(5) $${NSI}_{ij}=0.5\times {CVscore}_{ij}+0.5\times {RDscore}_{ij}$$

where NSI_ij_ represents the nutrient stability index of nutrient i in month j. Equal weights of 0.5 and 0.5 were assigned to CVscore and RDscore because within-period stability and representativeness relative to the annual mean were considered equally important for selecting a suitable diagnostic sampling period. Alternative weighting schemes were not tested in the present study because independent yield, quality, or fertilisation-response data were not available to objectively optimise the weights. Consequently, the NSI should be regarded as a preliminary exploratory index, with future studies potentially refining its weighting scheme by the integration of fertilisation gradients, yield performance, and plant nutrient status. The monthly NSI was obtained by averaging NSI_ij_ across nutrient elements:

(6) $${NSI}_{j}=\frac{1}{n}\sum_{i=1}^{n}{NSI}_{ij}$$

where NSI_j_ represents the integrated nutrient stability index of month j, and n represents the number of nutrient elements included in the calculation. NSI values were calculated for all nutrients, macronutrients (N, P, K, Ca, and Mg), and micronutrients (Fe, Mn, Zn, and Cu). Higher NSI values indicate greater nutrient stability and stronger representativeness of the corresponding sampling month.

### 2.5. Statistical and Multivariate Analyses

Data were expressed as mean ± standard deviations based on three biological replicates. Differences in nutrient concentrations among phenological stages were analysed using one-way analysis of variance (ANOVA), followed by Fisher’s least significant difference (LSD) test at *p* < 0.05. Pearson correlation analysis was performed to evaluate pairwise relationships among nutrient elements.

In addition to absolute nutrient concentrations, selected nutrient ratios were calculated to evaluate temporal changes in leaf nutrient balance. The ratios included N:P, N:K, K:Ca, Ca:Mg, Fe:Mn, and Zn:Cu. These ratios were calculated for each biological replicate and then averaged for each sampling month.

Principal component analysis (PCA) was performed to characterize multivariate changes in leaf nutrient composition across phenological stages. The concentrations of the nine nutrient elements were centered and scaled before PCA because macronutrients and micronutrients were expressed in different units and ranges. The PCA biplot was used to visualize the distribution of samples and the contribution of individual nutrients to the principal components.

To further visualize temporal patterns in nutrient composition, a hierarchical clustering heatmap was generated using monthly mean nutrient concentrations. Before heatmap construction, values were standardized by row using Z-score transformation, so that the color scale represented the relative increase or decrease of each nutrient across sampling months rather than differences in absolute concentration units. A separate heatmap was also generated for nutrient ratios. In this ratio heatmap, colors indicate row-wise Z-scores, whereas numbers in cells represent monthly mean nutrient ratios.

All statistical analyses and visualizations were performed in R version 4.4.2.

## 3. Results

### 3.1. Dynamic Changes of Macronutrient Content in P. ostii Leaves

**Figure 2:**
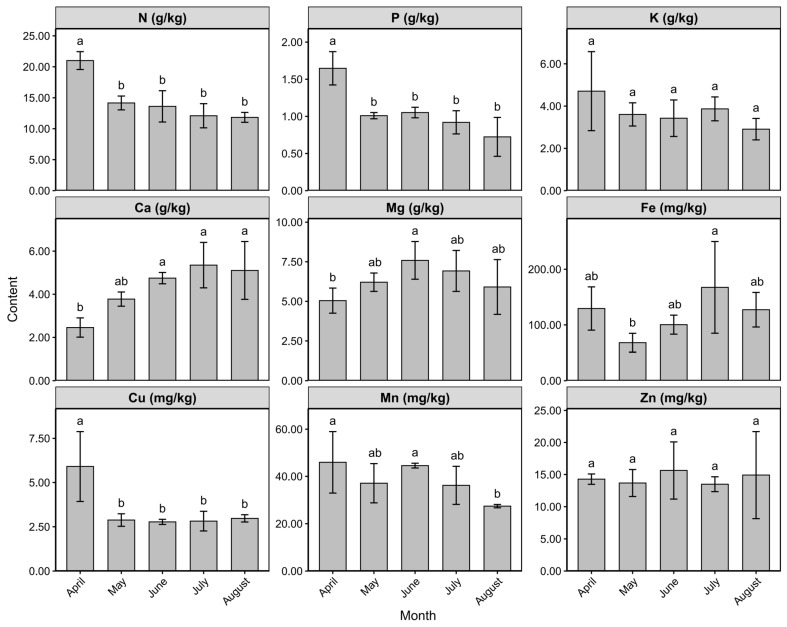
Seasonal changes in leaf nutrient concentrations of *P. ostii* at different phenological stages. Values are means ± standard deviations, *n* = 3. Different lowercase letters indicate significant differences among phenological stages for the same nutrient element according to one-way ANOVA followed by Fisher’s LSD test at *p* < 0.05.

The seasonal changes in leaf macronutrient concentrations are shown in Figure 2. The annual mean concentrations were found to be in the following order: N > Mg > Ca > K > P.

The concentrations of N and P in leaves showed analogous seasonal patterns, with the highest values observed in April during the flowering stage. The N concentration reached 21.01 g/kg in April and then decreased significantly from May onwards, remaining at a relatively stable level from May to August. In a similar manner, the P concentration exhibited a maximum of 1.65 g/kg in April, after which it underwent a substantial decline. No significant differences in N or P concentrations were observed among the months of May, June, July, and August. In contrast to the N and P contents, the K content exhibited no significant difference throughout the entire growth and development period (*p* > 0.05).

Ca and Mg showed different seasonal patterns from N and P. Mg increased from 5.05 g/kg in April to a maximum of 7.56 g/kg at fruit expansion in June, and then declined slightly. Ca increased progressively and reached its highest value of 5.35 g/kg in July, followed by a slight decline during foliar senescence.

### 3.2. Micronutrients Content Changes in Leaf Blades

The annual average content of micronutrients in *P. ostii* leaves was as follows: Fe > Mn > Zn > Cu. Specifically, the Fe concentration was 129.42 mg/kg at the flowering stage (April), decreased to 68.00 mg/kg at the initial fruit set stage (May), and then gradually increased to 167.42 mg/kg at the late fruiting stage (July). The Fe concentration in July exhibited a significant increase in comparison with that in May (*p* < 0.05), whereas April, June, and August showed intermediate values. The maximum Mn content in the leaves was 45.96 mg/kg during the flowering stage. Subsequently, the Mn content exhibited a substantial decline, reaching 27.42 mg/kg during the initial foliar senescence stage (*p* < 0.05). The Cu content in the leaves was 5.91 mg/kg at flowering and then declined significantly by 51.27% to 2.88 mg/kg at the initial fruit set (*p* < 0.05), remaining stable from May to August. Zn concentrations exhibited only marginal variation across the developmental stages (*p* > 0.05).

### 3.3. Evaluation of Diagnostic Sampling Periods in P. ostii

#### 3.3.1. Analysis of Coefficients of Variation and Deviations from Annual Means for Macronutrients and Micronutrients

Table 1 summarizes the temporal stability of nutrient concentrations in *P. ostii* leaves by ranking the coefficients of variation (CV) and the absolute deviations from the annual mean across growth stages. The combined rankings indicated that May was the optimal diagnostic period for N and Mg, May and July for K, May and June for P, and June for Ca. For micronutrients, the integrated analysis showed that August was the optimal diagnostic period for Fe and Cu, April for Zn, and June for Mn.

#### 3.3.2. Comprehensive Analysis of Coefficients of Variation and Deviation from Annual Mean Difference

For macronutrients, the summed CV ranks were highest in May and lowest in August, whereas the summed deviation ranks were highest in May and lowest in April (Table 2). When both metrics were integrated, the overall ranking of sampling periods was May > June > July > August > April.

The total CV analysis rankings for the micronutrients were as follows: June > August > May > July > April. Meanwhile, the deviation scores were ranked as follows: August > May > April > July > June. Integrating both the CV and deviation scores resulted in the following overall ranking: August > June > May > April = July (Table 3).

In summary, May and June were identified as relatively suitable periods for macronutrient diagnosis, while August and June showed higher suitability for micronutrient assessment. Because August coincided with early foliar senescence, June appeared to provide a better balance between nutrient stability and active plant growth. This result was further evaluated using the integrated nutrient stability index and multivariate analyses described below.

### 3.4. Correlation of Leaf Mineral Nutrient Content

**Figure 3:**
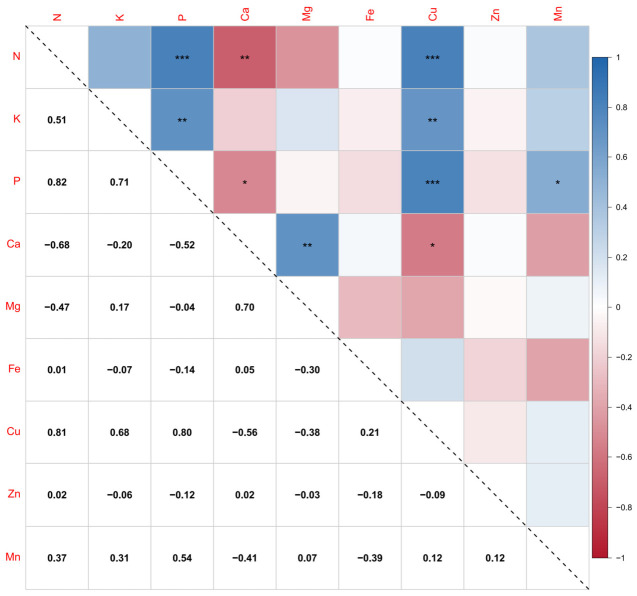
Correlations among nutrient elements in *P. ostii* leaves. The numbers represent the correlation coefficients between various traits. Different colors represent the magnitudes of the correlation coefficients between traits. * indicates a significant correlation at the 0.05 level. ** indicates a significant correlation at the 0.01 level. *** indicates a significant correlation at the 0.001 level.

Significant positive correlations were observed between N and P (*p* < 0.001, r = 0.82), N and Cu (*p* < 0.001, r = 0.81), K and P (*p* < 0.01, r = 0.71), K and Cu (*p* < 0.01, r = 0.68), P and Cu (*p* < 0.001, r = 0.80), P and Mn (*p* < 0.05, r = 0.54), and Ca and Mg (*p* < 0.01, r = 0.70). Negative correlations were detected between N and Ca (*p* < 0.01, r = −0.68), P and Ca (*p* < 0.05, r = −0.52), and Ca and Cu (*p* < 0.05, r = −0.56) (Figure 3).

### 3.5. Multivariate Nutrient Patterns and Stability Assessment

**Figure 4:**
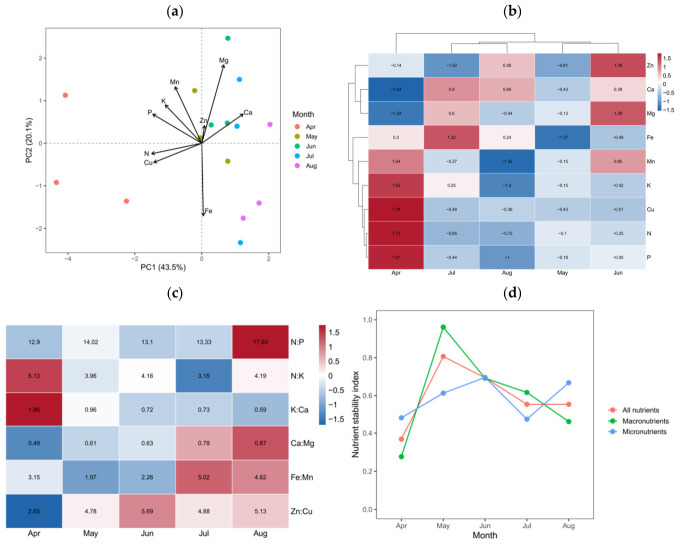
Multivariate characterization of leaf nutrient dynamics and nutrient stability in *P. ostii*. (**a**) PCA biplot based on standardized concentrations of nine nutrient elements. Points indicate biological replicates, and arrows indicate nutrient loading vectors. (**b**) Hierarchical clustering heatmap of monthly mean nutrient concentrations based on row-wise Z-scores. (**c**) Heatmap of selected nutrient ratios. Colors indicate row-wise Z-scores, and numbers in cells represent monthly mean ratios. (**d**) Nutrient stability index (NSI) for all nutrients, macronutrients, and micronutrients across sampling months. Higher NSI values indicate greater nutrient stability and representativeness.

Principal component analysis was performed to characterize the multivariate nutrient profile of *P. ostii* leaves across phenological stages. The first two principal components explained 63.6% of the total variance, with PC1 and PC2 accounting for 43.5% and 20.1%, respectively (Figure 4a). The April samples were clearly separated from most samples collected at later stages along PC1, indicating a shift in leaf nutrient composition from flowering to subsequent fruit development and foliar senescence. The loading vectors showed that N, P, K, Cu, and Mn were positioned toward the early-season side of PC1, whereas Ca and Mg were associated with fruit development and later-stage nutrient profiles. Fe showed a distinct loading direction from most other nutrients, consistent with its relatively strong temporal fluctuation during the growth cycle.

The Z-score heatmap based on monthly mean nutrient concentrations further revealed coordinated temporal patterns among nutrient elements (Figure 4b). N, P, K, and Cu showed relatively high values in April, corresponding to the flowering stage, whereas Ca and Mg were relatively higher during fruit development and later growth. Zn showed moderate temporal variation, while Fe displayed stronger fluctuation among months. These patterns indicate that seasonal nutrient changes in *P. ostii* leaves were not limited to isolated changes in individual elements, but reflected coordinated shifts in nutrient composition across phenological stages.

Nutrient ratio analysis further demonstrated marked changes in leaf nutrient balance during the growth cycle (Figure 4c). The N:P ratio increased from 12.90 in April to 17.93 in August. The N:K ratio was highest in April and showed lower values during the subsequent fruit development stages. The K:Ca ratio decreased from 1.86 in April to 0.59 in August, whereas the Ca:Mg ratio increased from 0.49 to 0.87 during the same period. In addition, Fe:Mn was relatively high in July and August, while the Zn:Cu ratio increased after flowering, from 2.65 in April to 4.78–5.69 from May to August. These results indicate a transition from an early-season nutrient balance characterized by relatively high N, P, K, and Cu status toward later-stage changes associated with Ca accumulation and micronutrient-ratio adjustment.

The nutrient stability index provided an integrated assessment of nutrient stability and representativeness across sampling periods (Figure 4d). For all nutrients combined, May showed the highest NSI, followed by June, indicating that these two months had relatively stable and representative leaf nutrient profiles. For macronutrients, May showed the highest stability, whereas for micronutrients, June and August showed relatively high NSI values. However, August corresponded to early foliar senescence, which reduces its practical suitability for routine nutrient diagnosis. Therefore, considering both nutrient stability and phenological relevance, June was regarded as a practical preliminary sampling window for comprehensive foliar nutrient diagnosis in *P. ostii* under the present experimental conditions.

## 4. Discussion

### 4.1. Annual Changes of Macronutrients in P. ostii Leaves

Many studies have analysed annual variations in leaf nutrient elements to guide fertilisation practices in a rational manner and enhance crop yield and quality [28,29]. While previous studies have established nutrient cycling patterns in fruit trees such as *Zizyphus jujube* [30] and *Prunus armeniaca* [31], there is a paucity of data for *P. ostii*.

The levels of N and P in *P. ostii* exhibited a peak at anthesis, followed by a significant decrease at the initial fruit set stage, after which they remained relatively stable. This decline is indicative of the elevated metabolic cost associated with reproductive development. Specifically, processes such as floral organogenesis and effective pollination are nutrient-intensive [32], suggesting the importance of adequate nutrient supply before and during flowering. Once fruits are set, the allocation of nutrient to developing fruits may increase, thus rendering the early fruit-setting stage in May is a critical nutrient allocation bottleneck. Despite the reapplication of N-P-K at this time, foliar N and P levels declined significantly, a response also documented by Wei et al. [21] and Xu et al. [33]. The substantial decrease in foliar N and P levels from April to May may be indicative of rapid nutrient allocation to reproductive organs during the processes of flowering and early fruit development. Therefore, future fertilisation regimes may consider adjusting the timing of the latter split to better coincide with the transition from flowering to initial fruit set. The K content in this study ranged from 2.91 g/kg to 4.71 g/kg, with no significant changes observed across different periods. This finding is consistent with the study on *Ilex vomitoria* by Zou Xue [34], but contrasts with the K dynamics reported for *Malus pumila* in the study by Wen [35].

Ca and Mg are critical macronutrients, each of which plays a distinct cellular role. As demonstrated in the relevant literature, Ca maintains cell-wall integrity and functions in signal-transduction cascades, necessitating continuous accumulation throughout the processes of leaf and fruit expansion [36]. In this study, the levels of Ca in *P. ostii* leaves increased progressively from the flowering stage to the late fruit maturation stage, with the highest value observed in July. This finding is consistent with the observations reported by Zhang et al. [37]. After July, a slight numerical decline was exhibited by Ca during the process of foliar senescence, a phenomenon that may be associate with its low phloem mobility and irreversible sequestration in pectic gels and oxalate crystals [38]. Mg is the central atom of chlorophyll and a cofactor for photosynthetic phosphorylation, and it is imperative that it is maintained at high levels during active growth [39]. Consequently, the Mg content of the leaves must be kept high during the plant’s growth and development. In the present study, Mg levels increased until the fruit expansion phase and then declined slightly. This finding is consistent with the observations reported by Li et al. [40] and may be associated with the heat sensitivity of *P. ostii*, whereby elevated temperatures could induce premature dormancy and reduce Mg retention in leaves.

### 4.2. Annual Dynamics of Micronutrients in P. ostii Leaves

The present study established that the highest concentrations of micronutrients in *P. ostii* leaves were found to be Fe, followed by Mn, Zn and Cu. This finding was consistent with the results reported by Liu et al. [41]. Fe primarily fulfils two core functions: electron transport during the light reaction and chlorophyll synthesis [42]. It is evident that Fe is a pivotal micronutrient for the effective progression of photosynthesis and chlorophyll biosynthesis in plants. In this study, foliar Fe concentrations exhibited a fluctuating trend, characterized by an initial decrease at the initial fruit-set stage, followed by an increase, with the highest value observed during late fruit development. This finding is similar to the results of research by Liu et al. on *Sapindus mukorossi* [43].

It is imperative to acknowledge the pivotal role of Cu in facilitating the successful reproduction of plants. This essential element plays a key role in supporting the development of floral organs, with its demand reaching its zenith during the flowering stage [44]. After the initial stage of fruit set, the floral organs gradually undergo senescence, a process that may be accompanied by a decline in Cu demand [45]. In this study, the Cu content decreased during the sampling process, with the highest Cu content being observed during the flowering stage. This finding is consistent with the conclusion of Liu et al. [41] that flowering is a critical period for foliar Cu acquisition.

Mn is a crucial micronutrient for plant photosynthesis, playing a primary role in the light reaction stage. It functions not only as a constituent of the oxygen-evolving complex (OEC) in photosystem II (PSII), facilitating water photolysis and maintaining the structural stability of PSII and the efficient operation of photosynthetic electron transport. Additionally, Mn also indirectly contributes to chlorophyll synthesis and light energy utilisation [46]. In the context of the present study, the Mn content showed a trend of decrease, increase, and subsequent decrease during the primary developmental process, with a substantial accumulation occurring during the fruit expansion phase. This phenomenon is analogous to the results observed in *Actinidia chinensis*, which also exhibits relatively high Mn content during the fruit expansion phase [47]. The relatively elevated Mn levels observed during fruit expansion may be related to the physiological activity of leaves at this particular stage. However, further investigation is required to elucidate the underlying mechanism.

As a structural component of enzymes involved in processes such as chlorophyll biosynthesis, Zn is required to maintain basic physiological functions throughout plant development [48]. Such a steady demand would not lead to dramatic fluctuations in its foliar content. In accordance with this consistent demand, the data demonstrated no significant changes in Zn content in *P. ostii* leaves throughout the sampling period. Similarly to the present study, Ren et al. [49] found that the leaves of *Artocarpus heterophyllus* showed no obvious peak in Zn content throughout the whole year.

### 4.3. Sampling Time Definition of P. ostii

Given the significant impact of fluctuations in developmental stage on the outcomes of foliar nutrient diagnosis, the timing of sampling is of critical importance for achieving accurate results [50]. In this study, the optimal sampling time for nutritional diagnosis was determined by scoring mean differences and coefficients of variation. The results indicated that N, P, K, Ca, Mg, Cu, and Mn exhibited maximal mean differences during flowering, while K, Mn, Cu, and Fe showed elevated coefficients of variation. The combination of these metrics resulted in the lowest composite scores, thereby identifying the flowering phase as the least suitable diagnostic period. This instability is hypothesised to arise from the prioritized allocation of nutrients to the processes of floral organogenesis and pre-bud differentiation, in conjunction with accelerated nutrient translocation rates that amplify elemental fluctuations. A similar study by Mo et al. [18] also observed these patterns in *Camellia oleifera*, where intense nutrient competition during flower bud differentiation caused pronounced leaf nutrient fluctuations and reduced diagnostic reliability.

It is important to note that different plant species have different most suitable diagnostic times. For instance, *Artocarpus heterophyllus* attains diagnostic stability during the pre-floral bud differentiation phase, a stage distinguished by minimal elemental variability and maximal nutrient sink activity [27]. Conversely, *Mangifera indica* requires sampling in December during the period of active reproductive growth [51]. In *P. ostii*, May ranked highest for macronutrients and August ranked highest for micronutrients based on the integrated scoring of coefficients of variation and deviations from the annual mean. The NSI analysis further indicated that May and June exhibited relatively stable and representative leaf nutrient profiles, while August also showed relatively high micronutrient stability. However, it should be noted that August coincided with pod maturation and foliar senescence, which reduces its practical value for routine diagnosis. In consideration of the stability of nutrients, the active growth of plants, and the phenological relevance, the month of June was determined to provide the best overall balance. Consequently, it is recommended that June be utilised as a preliminary sampling window for the diagnosis of foliar nutrients under the conditions established in this study. Concurrently, May may offer an earlier opportunity for macronutrient diagnosis, as macronutrients play important roles in fruit set and early fruit development. This earlier diagnostic period may facilitate the adjustment of fertilisation practices by growers prior to the onset of fruit expansion. The findings of this study indicate that the June sampling window is applicable to eight-year-old trees at full fruiting capacity. However, further validation is required to ascertain the applicability of this window to younger or older trees.

### 4.4. Interrelationships of Nutrient Elements in P. ostii

It has been reported that significant positive or negative correlations between mineral elements in different plant organs may indicate synergistic or antagonistic interactions, respectively, of the nutrients analyzed [52]. In the present study, correlation analysis was employed to reveal multiple associations among nutrients in *P. ostii* leaves. These included positive correlations between N and P, N and Cu, K and P, K and Cu, P and Cu, P and Mn, and Ca and Mg, as well as negative correlations among N and Ca, P and Ca, and Ca and Cu. The positive correlations between N and Cu, and between P and Cu, may primarily reflect their analogous seasonal patterns in *P. ostii* leaves. The levels of these nutrients were found to be elevated during the flowering stage and subsequently decreased after fruit set, indicating a potential role for these nutrients in the elevated metabolic demand that occurs during the early stages of reproductive development. However, correlation does not necessarily indicate a direct physiological dependence. Therefore, Cu status should still be evaluated independently in foliar nutrient diagnosis, rather than being inferred from N or P concentrations alone. These relationships suggest that nutrient uptake, transport, and redistribution in *P. ostii* leaves are coordinated rather than independent during seasonal development. These correlations parallel those observed in *Durio zibethinus*, where P-K synergy and Ca-Mg facilitation were documented [53], as well as in forest ecosystems where N-P covariation is widespread [54].

The nutrient ratio analysis provided further evidence for changes in nutrient balance during the growth cycle. The increase in N:P toward foliar senescence was mainly associated with a stronger decline in P than in N, suggesting a relative shift in N-P balance during late growth. The decline in K:Ca, together with the increase in Ca:Mg, reflected the progressive accumulation of Ca during fruit development and later growth. Because Ca is closely associated with cell-wall stability, membrane function, and structural development, this shift may indicate an increasing structural nutrient demand during fruit and leaf maturation. In addition, the increase in Zn:Cu after flowering was largely related to the sharp decline in Cu, which is consistent with the high Cu demand during flowering and reproductive organ development.

Collectively, these results indicate that the selection of an appropriate sampling period is essential for reliable foliar nutrient diagnosis in *P. ostii*. The diagnostic window should not be determined exclusively by the absolute concentration of individual elements, but should also consider the nutrient balance, the phenological nutrient demand, and the stability of leaf nutrient profiles. Under the conditions of this study, June was identified as a practical diagnostic window, owing to the convergence of relatively stable nutrient concentrations and the fruit expansion stage. The coordinated changes among N, P, K, Cu, Ca, and Mg further suggest that nutrient diagnosis at this stage can provide useful information for subsequent fertilisation decisions. Specifically, the application of N and P should be given particular consideration flowering and during the early stages of fruit development. In contrast, the application of K should be adjusted according to foliar or soil diagnosis because no significant seasonal change was observed. Ca and Mg should be monitored during fruit expansion and late fruit development, whereas Cu should receive attention during flowering. Fe and Mn should be managed according to foliar diagnostic results, and Zn supplementation may only be necessary when deficiency is detected. These recommendations are qualitative and should be interpreted as diagnostic guidance rather than direct fertiliser-rate prescriptions. Future fertilisation experiments with different application rates are needed to validate these recommendations.

### 4.5. Limitations and Future Perspectives

Despite the identification of June as a practical sampling window for foliar nutrient diagnosis in *P. ostii*, it is imperative to acknowledge the limitations of this study. Firstly, the study was conducted over the course of a single year and at a single experimental site, with the study focusing on eight-year-old trees at full fruiting capacity. Therefore, the recommended June sampling window may be particularly applicable to full-fruiting *P. ostii* trees under similar cultivation conditions, but its broader applicability still requires further validation. Different soil types, tree ages, cultivars, and climatic conditions may affect nutrient dynamics and the optimal diagnostic period. Secondly, although the basic chemical properties of the soil at the experimental location were determined, the dynamic relationships between soil nutrient availability and leaf nutrient concentrations during different phenological stages were not quantified. In addition, soil trace elements, including Fe, Mn, Cu, and Zn, were not measured in the present study, which may limit the interpretation of soil–leaf relationships for micronutrients. Thirdly, only leaf samples were collected; therefore, nutrient accumulation in roots, stems, flowers, fruits, and seeds was not analysed, and the bioconcentration factor and translocation factor could not be calculated. Future studies should integrate multi-year and multi-site validation with soil nutrient analysis and multi-organ nutrient measurements to clarify nutrient uptake, accumulation, and translocation patterns in *P. ostii*. To establish reliable foliar nutrient reference values and to develop more precise fertilisation recommendations, larger datasets from different orchards and growing seasons are also required.

## 5. Conclusions

The concentrations of nutrients present within the leaves of *P. ostii* exhibited significant variation across the various phenological stages. The levels of N, P, and Cu were found to be relatively high during the flowering stage, whereas Ca and Mg levels increased during fruit development and subsequent growth. Multivariate analyses further revealed coordinated changes in leaf nutrient composition and nutrient balance. Based on the combined evaluation of the CV, deviations from the annual mean, and the NSI, May and June were relatively stable periods for the assessment of leaf nutrient content. In consideration of the nutrient stability and the phenological relevance, the month of June, corresponding to the fruit expansion stage, may serve as a practical sampling window for foliar nutrient diagnosis under the conditions of this study. These findings contribute to the establishment of an appropriate sampling window for foliar nutrient diagnosis, thereby providing a foundation for the development of nutrient monitoring and future fertilisation studies in *P. ostii*.

## Figures and Tables

**Table 1:** Temporal stability and representativeness of leaf nutrient concentrations in *P. ostii* across different months.

Nutrient Element	Month	Mean	SD	CV(%)	CV Rank	Deviation from Annual Mean	Deviation Rank	Comprehensive Score
N	April	21.01	1.44	6.87	4	6.47	1	5
N	May	14.16	1.12	7.89	3	0.38	5	8
N	June	13.61	2.53	18.57	1	0.93	4	5
N	July	12.09	1.95	16.14	2	2.45	3	5
N	August	11.84	0.80	6.77	5	2.70	2	7
P	April	1.65	0.22	13.62	3	0.58	1	4
P	May	1.01	0.04	4.16	5	0.06	4	9
P	June	1.05	0.07	6.75	4	0.02	5	9
P	July	0.92	0.16	16.97	2	0.15	3	5
P	August	0.72	0.26	36.18	1	0.35	2	3
K	April	4.71	1.87	39.71	1	1.00	1	2
K	May	3.61	0.55	15.24	4	0.10	5	9
K	June	3.43	0.86	25.23	2	0.28	3	5
K	July	3.87	0.57	14.61	5	0.17	4	9
K	August	2.91	0.51	17.43	3	0.80	2	5
Ca	April	2.46	0.45	18.18	3	1.83	1	4
Ca	May	3.78	0.33	8.73	4	0.51	4	8
Ca	June	4.75	0.26	5.52	5	0.46	5	10
Ca	July	5.35	1.05	19.70	2	1.06	2	4
Ca	August	5.10	1.34	26.27	1	0.82	3	4
Mg	April	5.05	0.79	15.72	3	1.29	1	4
Mg	May	6.21	0.58	9.35	5	0.13	5	10
Mg	June	7.56	1.19	15.69	4	1.25	2	6
Mg	July	6.92	1.29	18.69	2	0.59	3	5
Mg	August	5.91	1.73	29.28	1	0.43	4	5
Fe	April	129.42	38.89	30.05	2	10.92	4	6
Fe	May	68.00	16.88	24.82	3	50.50	1	4
Fe	June	100.42	17.08	17.00	5	18.08	3	8
Fe	July	167.42	82.38	49.20	1	48.92	2	3
Fe	August	127.25	31.05	24.40	4	8.75	5	9
Cu	April	5.91	1.98	33.50	1	2.44	1	2
Cu	May	2.88	0.35	12.28	3	0.59	4	7
Cu	June	2.78	0.14	5.21	5	0.70	2	7
Cu	July	2.82	0.56	19.70	2	0.65	3	5
Cu	August	2.97	0.21	6.91	4	0.50	5	9
Zn	April	14.28	0.80	5.62	5	0.12	5	10
Zn	May	13.68	2.10	15.34	3	0.72	3	6
Zn	June	15.63	4.45	28.48	2	1.23	1	3
Zn	July	13.49	1.15	8.55	4	0.91	2	6
Zn	August	14.92	6.78	45.42	1	0.52	4	5
Mn	April	45.96	13.02	28.33	1	7.73	2	3
Mn	May	37.10	8.29	22.36	2	1.13	5	7
Mn	June	44.55	1.02	2.29	5	6.32	3	8
Mn	July	36.20	8.05	22.23	3	2.03	4	7
Mn	August	27.42	0.69	2.52	4	10.81	1	5

Note: Mean and SD values for N, P, K, Ca, and Mg are expressed in g/kg, whereas Fe, Cu, Zn, and Mn are expressed in mg/kg.

**Table 2:** Comprehensive analysis of monthly difference scores for macronutrients in *P. ostii* leaves.

Difference Score	April	May	June	July	August
CV Rank	14	21	16	13	11
Deviation Rank	5	23	19	15	13
Comprehensive Score	19	44	35	28	24

**Table 3:** Comprehensive analysis of monthly difference scores for micronutrients in *P. ostii* leaves.

Difference Score	April	May	June	July	August
CV Rank	9	11	17	10	13
Deviation Rank	12	13	9	11	14
Comprehensive Score	21	24	26	21	27

## Data Availability

All data generated or analysed during this study are included in this published article.
